# Medical News

**Published:** 1853-11

**Authors:** 


### MEDICAL NEWS.

HOSPITAL APPOINTMENTS.—Dr. WILLARD PARKER has resigned the situation of Visiting Surgeon to the Bellevue Hospital, N. Y., and Dr. LEWIS A. SAYRE has been appointed to fill the vacancy.

Dr. WILLIAM HUNT has been appointed one of the Surgeons to the Hospital of the Protestant Episcopal Church of this city, to succeed Dr. W. B. PAGE, resigned; and Dr. DEACON has been appointed one of the Physicians to this Institution, to succeed Dr. F. G. SMITH, resigned.

Surgeon WILLIAM WHEELER, U. S. N., has been appointed Chief of the Bureau of Medicine and Surgery, vice Surgeon THOMAS HARRIS.

SAVANNAH MEDICAL COLLEGE.—This Institution will go into operation on the first Monday in November. The course of lectures will be delivered by R. D. Arnold, on the Theory and Practice of Medicine; Dr. G. M. Kollock, on Obstetrics and the Diseases of Women and Children; Dr. W. G. Bulloch, on the Principles and Practice of Surgery; Dr. C. W. West, on Medical Chemistry; Dr. H. L. Byrd, on Materia Medica and Therapeutics; Dr. E. H. Martin, on Physiology; Dr. J. G. Howard, on Anatomy; Dr. J. B. Read, Demonstator of Anatomy, on Pathological Anatomy.

Dr. TINSLEY, of Cuba, claims to have discovered, that "vaccine virus, after passing through a negro's system, is valueless for the white race."

COMPULSORY VACCINATION IN GREAT BRITAIN.—The new Act to extend and make compulsory the practice of vaccination, provides that parishes or unions, if need be, are, within six weeks of the passing of this Act, to be divided into districts, for the purpose of vaccination, and places are to be appointed for performance of the operation. The parents and guardians of children born after the 1st of August, 1853, are to have such children vaccinated within three or four months after birth, and the children are to be taken for the inspection of the medical officer on the eighth day after the operation, to ascertain the result of the same. Should the parents neglect to have the child vaccinated, they are liable to a penalty not exceeding twenty shillings.

OBITUARY.—Dr. W. COCHRAN, one of the oldest and most respected physicians of Louisville, Ky., died at Biloxi, Miss., on the 20th of August last.

DEATH OF MR. BRANSBY B. COOPER.—Bransby Blake Cooper, Esq., F. R. S., Senior Surgeon to Guy's Hospital, and nephew to the late Sir Astley Cooper, died in London, on the 11th of August. Mr. Cooper was born September 2, 1792; he first served as assistant surgeon in the artillery, studied for some time at Edinburgh, and was eventually apprenticed to Sir Astley Cooper, his uncle, under whose fostering care, and by his own exertions and abilities, he gained the position of senior surgeon to one of the first institutions of the metropolis.

Mr. Cooper was the author of Lectures on Surgery, and was distinguished as a good writer and lecturer, a skilful surgeon and operator, and a laborious practitioner, until recently his exertions were necessarily relaxed by the onset and continuance of disease. When he first experienced the sensation of ulceration at the pharynx, he believed that he had swallowed some small bone, which had lacerated the parts, or was adhering to the tongue or pharynx. Several of Mr. Cooper's friends and colleagues were requested to inspect the throat, with a view to the discovery of the foreign body; but, as may be readily imagined, no extraneous substance was found, and the ulceration continued to make progress; the patient becoming at the same time emaciated, and considerably debilitated. As we stated above, Mr. Cooper continued, in spite of his sufferings, to pay occasional visits to Guy's where he was an object of great sympathy to his colleagues and the pupils, who had always been treated by him in so kind and friendly a manner that they could not but feel much attachment for their respected friend and teacher. A small vessel (as proved by the autopsy) gave way in the posterior lingual region whilst Mr. Cooper was at the Athenæum Club, and he was almost instantly suffocated.

This eminent surgeon and amiable man was buried on the 25th Aug. when his remains were accompanied to the family vault, in St. Martin's Church, by a few relations, his colleagues at Guy's, and a limited number of students. All seemed to be impressed with great sympathy for the departed in paying this tribute of respect to the kind man, the valued teacher, and accomplished surgeon.

### CIRCULAR OF INSTRUCTIONS TO SPECIAL EXAMINERS OF DRUGS.

*To prevent the Importation of Adulterated Drugs and Medicines.*

To Collectors and other Officers of the Customs, under the Act of 26th June, 1848.

Treasury Department, June 4, 1853.

"It being represented to this Department that much embarrassment has been experienced by officers of the customs, at some of the ports of the United States, in reference to the provisions of the act of 26th June, 1848, 'to prevent the importation of adulterated and spurious drugs and medicines,' it is deemed expedient, with a view to avoid future difficulties arising from misconstruction of the law, and to secure uniformity of practice at the several ports in carrying out its provisions with precision and efficiency, to furnish you with the additional instructions which follow, explanatory and in modification of the circular instructions addressed to you by the department on the 7th July, 1848.

"To avoid the recurrence of a difference of opinion between the officers of the customs as to what particular articles of commerce should be considered drugs and medicines, and as such, be subject to special examination by the special examiner of drugs and medicines, it is thought proper to state that, in conformity with the evident spirit and intent of the law, it is required that all articles of merchandize used wholly or in part as medicines, and found described as such in the standard works specially referred to in the act, must be considered drugs and medicines, and that all invoices, therefore, of such articles, in whole or in part, must be submitted to the special examination of the special examiner of drugs and medicines, before they can be permitted to pass the custom house.

"In the examination on entry of any medicinal preparation, the said special examiner is to unite with the appraiser.

"With a view to afford a reliable guide to the examiner of drugs and medicines, as well as to the analytical chemist, on appeal, in ascertaining the admissibility of such articles under the provision of the law founded on their purity and strength, the following list is given of some of the principal articles, with the result of special tests agreeing with the standard referred to in the law, all of which articles are to be entitled to entry when ascertained by analysis to be composed as noted, viz:

"Aloes, when affording 80 per cent. of pure aloetic extractive.

"Assafœtida, when affording 50 per cent. of its peculiar bitter resin, and 3 per cent. of volatile oil.

"Cinchona Bark, when affording one per cent. of pure quinine, whether called Peruvian, Calisaya, Arica, Carthagena, Maracaibo, Santa Martha, Bogota, or under whatever name, or from whatever place; or

"Cinchona Bark, when affording 2 per cent. of the several alkaloids combined, as quinine, cinchonine, quinidine, aricine, &c., the bark of such strength being admissible as safe and proper for medicine and useful for chemical and manufacturing purposes.

| | | | | |
|---|---|---|---|---|
| Benzoin, | when affording | 80 | per cent. | of resin, or |
| " | " | 12 | " | of benzoic acid. |
| Colocynth, | " | 12 | " | of colocynthin. |
| Elaterium, | " | 30 | " | of elaterin. |
| Galbanum, | when affording | 60 | " | of resin. |
| " | " | 10 | " | of gum and |
| " | " | 6 | " | of volatile oil. |
| Gamboge, | " | 70 | " | of pure gamboge resin, and |
| " | " | 20 | " | of gum, |
| Guaiacum, | " | 80 | " | of pure guaiac resin, |
| Gum ammoniac | " | 70 | " | of resin and |
| " | " | 18 | " | of gum. |

Jalap, when affording 11 per cent. of pure jalap resin, whether in root or powder.

| | | | | |
|---|---|---|---|---|
| Manna, | when affording | 37 | per cent. | of pure mannite. |
| Myrrh, | " | 30 | " | of pure myrrh resin, and |
| " | " | 50 | " | of gum. |
| Opium, | " | 9 | " | of pure morphine. |
| Rhubarb, | " | 40 | " | of soluble matter, whether in root or powder; none admissible but the articles known as East India, and Turkey or Russian rhubarb. |

Sagapenum, 50 per cent. of resin.
" 30 per cent. of gum, and
Sagapenum 2 per cent. of volatile oil.
Scammony, 70 per cent. of pure scammony resin.
Senna, 28 per cent. of soluble matter.

"All medicinal leaves, flowers, barks, roots, extracts, &c., not herein specified, must be imported, in perfect condition, and of as recent collec-lection and preparation as practicable.

"All pharmaceutical and chemical preparations whether crystallized or otherwise, used in medicine, must be found, on examination, to be pure and of proper consistence and strength, as well as of perfect manufacture, conformably with the formulas contained in the standard authorities named in the act; and must in no instance contain over three per cent. of excess of moisture or water of crystallization.

"Essential or volatile oils, as well as expressed oils, used in medicine, must be pure, and conform to the standards of specific gravity noted and declared in the dispensatories mentioned in the act.

"'Patent or secret medicines,' are by law subject to the same examination, and disposition after examination, as other medical preparations, and cannot be permitted to pass the Custom House for consumption, but must be rejected and condemned, unless the special examiner be satisfied, after due investigation, that they are fit and safe to be used for medicinal purposes.

"The appeal from the report of the special examiner of drugs and medicines, provided for in the act, must be made by the owner or consignee within ten days after the said report; and in case of such appeal, the analysis made by the analytical chemist is expected to be full and in detail, setting forth clearly and accurately, the name, quantity, and quality of the several component parts of the article in question; to be reported to the collector under oath or affirmation.

"On such report being made, a copy of the same will be immediately furnished by the collector to the special examiner of drugs and medicines, who, if the report be in conflict with his return made to the collector, and he have cause to believe that the appeal and analytical examination have not been conducted in strict conformity with the law, may enter his protest in writing against the reception and adoption by the collector of such report and analysis, until a reasonable time be allowed for the preparation of his views in the case, and their submission to this department for its consideration.

James Guthrie,

Secretary of the Treasury."

